# Dietary nitrate improves jaw bone remodelling in zoledronate‐treated mice

**DOI:** 10.1111/cpr.13395

**Published:** 2023-02-21

**Authors:** Wen Pan, Jianyu Gu, Shihan Xu, Chunmei Zhang, Jinsong Wang, Songlin Wang, Junji Xu

**Affiliations:** ^1^ Salivary Gland Disease Centre and Beijing Key Laboratory of Tooth Regeneration and Function Reconstruction, School of Stomatology, Beijing Laboratory of Oral Health Capital Medical University Beijing China; ^2^ Department of Biochemistry and Molecular Biology, School of Basic Medical Sciences Capital Medical University Beijing China; ^3^ Immunology Research Centre for Oral and Systemic Health, Beijing Friendship Hospital Capital Medical University Beijing China; ^4^ Laboratory for Oral and General Health Integration and Translation, Beijing Tiantan Hospital Capital Medical University Beijing China; ^5^ Research Units of Tooth Development and Regeneration, Chinese Academy of Medical Sciences Beijing China; ^6^ Department of Periodontics, Beijing Stomatological Hospital Capital Medical University School of Stomatology Beijing China; ^7^ Shanghai Stomatological Hospital & School of Stomatology Fudan University Shanghai China

## Abstract

Bisphosphonate‐related osteonecrosis of the jaw (BRONJ) is a serious complication that occurs in patients with osteoporosis or metastatic bone cancer treated with bisphosphonate. There is still no effective treatment and prevention strategy for BRONJ. Inorganic nitrate, which is abundant in green vegetables, has been reported to be protective in multiple diseases. To investigate the effects of dietary nitrate on BRONJ‐like lesions in mice, we utilized a well‐established mouse BRONJ model, in which tooth extraction was performed. Specifically, 4 mM sodium nitrate was administered in advance through drinking water to assess the short‐ and long‐term effects on BRONJ. Zoledronate injection could induce severe healing inhibition of the tooth extraction socket, while addition of pretreating dietary nitrate could alleviate the inhibition by reducing monocyte necrosis and inflammatory cytokines production. Mechanistically, nitrate intake increased plasma nitric oxide levels, which attenuated necroptosis of monocytes by downregulating lipid and lipid‐like molecule metabolism via a RIPK3 dependent pathway. Our findings revealed that dietary nitrate could inhibit monocyte necroptosis in BRONJ, regulate the bone immune microenvironment and promote bone remodelling after injury. This study contributes to the understanding of the immunopathogenesis of zoledronate and supports the feasibility of dietary nitrate for the clinical prevention of BRONJ.

## Graphical abstract

(A) Schematic overview of study procedures: 4 mM nitrate was added to the drinking water of C57/BL6 mice 1 week before injection of zoledronate up to 1 or 5 weeks after extraction of the right upper first molar. (B) Proposed model: Dietary nitrate could regulate monocyte necrosis stimulated by zoledronate by producing NO in vivo, thus regulating immunity and promoting bone remodelling after injury.

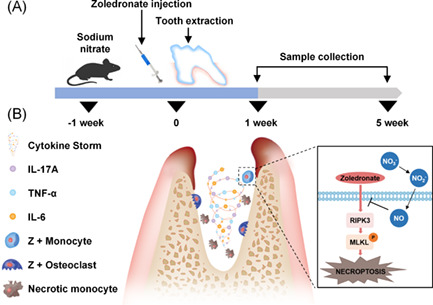

## 1 INTRODUCTION

Bisphosphonates‐related osteonecrosis of the jaw (BRONJ) was firstly described in medical literature in 2003.^1^ BRONJ, due to bone resorption inhibition, is reported as a side‐effect of bisphosphonates which are used widely for treating osteoporosis, bone metastases in cancer and some other skeletal‐related events. BRONJ is still one of the most common adverse events in stomatology,^2^ with multiple clinical reports indicating that dental procedures such as routine tooth extraction markedly increased the incidence of BRONJ. Although the osteoclast dysfunction theory and systemic inflammation may be partially involved,^2^, ^3^ the pathogenesis of BRONJ is not fully understood. And there is no effective treatment for BRONJ for now.

Nitric oxide (NO) can quickly diffuse through the membrane, serving as an important signalling molecule in biological events.^4^ Low concentrations of NO could regulate the polarization of macrophages and reduce the production of proinflammatory factors.^5^ It has been reported that NO is involved in the differentiation and activation of osteoclasts and osteoblasts, promoting proliferation at low concentrations and inhibiting proliferation at high concentrations.^6^ Inducible nitric oxide synthase (iNOS) plays an important role in the development and survival of osteoclasts in vivo. Only dysfunctional osteoclasts were induced in vitro by monocytes lacking iNOS. These results suggest that NO is essential for the differentiation and activation of osteoclasts.^6^, ^7^ However, the injection of bisphosphonates reduced iNOS expression, limiting the production of endogenous NO^8^, ^9^ and might aggravate the pathological changes. Therefore, the supplement of exogenous NO is essential.^10^

Sodium nitrate is omnipresent in our daily diet, especially in green vegetables, such as spinach, lettuce and beetroot. Previous studies have shown that sodium nitrate had protective effects in systemic diseases which were achieved by regulating the immune response and promoting and maintaining cell growth after injury.^11^, ^12^, ^13^ The molecular mechanisms behind it are complex and not fully understood. In the oral and gastrointestinal tract microenvironments, NO could be generated by commensal nitrate‐reducing bacteria through the nitrate‐nitrite‐NO axis.^14^ In contrast to NO synthases, the nitrate‐nitrite‐NO pathway is independent of L‐arginine and is unaffected by bisphosphonates.

In this study, the high‐prevalence of BRONJ in the animal model was used and modified as previously described.^15^, ^16^ The healing of tooth extraction sockets and their underlying mechanisms were explored after dietary nitrate supplementation. Our findings may have implications for the development of similar therapy for patients with BRONJ.

## 2 MATERIALS AND METHODS

### 2.1 Nitrate administration and BRONJ‐like lesion model

All experimental protocols and procedures were approved by the Institutional Animal Care and Use Committee of Capital Medical University (Ethics No. AEEI‐2021‐281). Female C57BL/6 J mice, aged 8 weeks,^17^ were obtained from Charles River Laboratories (Beijing, China).

Briefly, before tooth extraction, C57BL/6 J mice were intravenous injection of 600 μg/Kg zoledronate (Zometa, designated as Z; Novartis, Stein, Switzerland) as previously described.^15^, ^16^ No steroids were administered. The animals were grouped as follows: (1) the Ctl group, in which only the right maxillary first molars were removed; (2) Z group, which received zoledronate injection and tooth extraction; (3) Z + Nit group, which received zolendronate and 4 mM of sodium nitrate. The dose of 4 mM of sodium nitrate was repeated by our team for many times and was considered as the optimal concentration.^18^ Sodium nitrate 4 mM was added to the drinking water of mice once a week before zoledronate injection and continued until euthanasia. The maxillary first molar was extracted after the zoledronate injection. After tooth extraction, a stereomicroscope was used to confirm whether dental roots of the extracted molar were fractured. Mice with fractured roots were excluded in this study since the fractured roots affected wound healing following tooth extraction.

### 2.2 Flow cytometric analyses of tissue and peripheral blood

The tissue around the tooth sockets and peripheral blood were collected during the inflammatory phase of tissue 1 week after tooth extraction. Peripheral blood was processed by lysing the red blood cells. The tissue was digestion, centrifugation and filtration. Finally, the single cell suspension was stained for immune cell analysis. The cells were incubated with purified anti‐mouse CD45, anti‐mouse F4/80, anti‐mouse CD11b, anti‐mouse CD11c, anti‐mouse Ly6C, anti‐mouse Ly6G and anti‐mouse CD3 (Biolegend) at 4 °C for 30 min. Stained cells were tested on FACS Symphony (BD Biosciences), and data were analysed with FlowJo software.

### 2.3 Non‐targeted metabolomic profiling for monocytes from blood

The blood from the mice was collected by cardiac puncture. Then, monocytes were isolated by Ficoll‐Paque density gradient centrifugation and EasySep Mouse Monocyte Isolation Kit (Stemcell Technologies) according to the manufacturer's instructions.

Monocytes were ground using a tissue crusher, followed by sonication. After centrifugation and filtering, 20 μl of the supernatant was collected for liquid chromatography‐mass spectrometry (LC–MS) analysis. Metabolome profiling was performed using a Thermo Scientific Dionex ΜltiMate 3000 Rapid Separation LC system coupled with a Q Exactive hybrid quadrupole Orbitrap mass spectrometer (Thermo Scientific). Metabolites were analysed in the ESI+ and ESI‐modes. Data were collected and processed using Progenesis QI 2.3 (Waters Corporation, Milford, MA) software. Data analysis was performed by Beijing Qinglian Biotech, Co., Ltd.

### 2.4 Bone marrow‐derived monocyte isolation and culture conditions

Bone marrow‐derived monocytes were obtained from 8‐week‐old C57BL6J mice and seeded in 6‐well plates in RPMI 1640 medium containing 10% foetal bovine serum and 10 ng/ml murine M‐CSF at 37°C under 5% CO_2_. On the third day, 10 μM of zoledronate^19^ was added to the medium. Exogenous NO and NO scavenger were provided by 10 μM sodium nitroprusside (SNP) and 100 μM Carboxy‐PTIO (CP), respectively. After 24 h, monocytes were detected.

### 2.5 Microcomputed tomography (microCT) assessment

Occlusal view images of tooth extraction sockets were taken just after euthanasia with a stereomicroscope. Then, the maxilla was dissected, fixed with 4% paraformaldehyde, and scanned by SKyScan1276 (Bruker SkyScan, Aartselaar, Belgium). The parameters were set as follows: Source Voltage (kV) = 80, Source Current (μA) = 200, image pixel size (μm) = 7.042090, depth (bits) = 16, reference intensity = 58,000, exposure (ms) = 600 and rotation = Step (deg) = 0.400. Images were reconstructed using a three‐dimensionally reconstructed software program (NRecon and CTVox, Bruker).

To measure bone healing in the extraction socket, regions of interest (ROIs) were drawn in the hard tissue of tooth extraction sockets. For each socket, a cylinder containing the socket was set up, and the percentage of bone volume (bone volume/total tissue volume [%] and BMD [bone mineral density]) was measured (DataViewer and CTAn, Bruker). The imaging conditions and thresholds of CT were kept constant across all analyses. Two blinded investigators performed all measurements.

### 2.6 Validation of BRONJ‐like lesions by histomorphometry

After fixation of right maxillae, demineralization was performed with 10% EDTA at 4 °C for 2 months. Demineralized maxillae were paraffin‐embedded and sectioned at a thickness of 5 μm. HE, TRAP and Masson's trichrome staining were carried out. ROIs in the tooth extraction sockets were determined as follows: the area surrounded by the line linking the tops of the mesial and distal alveolar ridges, and the curve parallel to the line within 50 μm of the outer line of the laminar dura in the hard tissue of tooth extraction sockets. Moreover, the areas of connective tissue, excluding epithelium above the line linking the tops of the mesial and distal alveolar ridges, were also determined as ROIs in the soft tissue of tooth extraction sockets (Figure S2). The parameters were histomorphometrically assessed to validate impaired tooth extraction socket healing showing human‐like BRONJ conditions, as previously described.^20^, ^21^, ^22^

### 2.7 NOx levels in the plasma and tissue

Since NO can be rapidly oxidized to nitrate and nitrite and then released back as NO or other products in vivo,^23^ the concentration of NO cannot be measured directly in vivo. Therefore, the stable NO donors, nitrite and nitrate, and their end products reflect the total systemic NO concentration and can be used as a surrogate indicator of NO bioavailability. The combined measurement of NO metabolites (nitrate and nitrite) is referred to as NOx.^24^ The plasma and the supernatant from tooth sockets tissue were collected. Before assay, samples were filtered using 10,000 MW filters and diluted. Total Nitric Oxide and Nitrate/Nitrite Parameter Assay Kit (R&D) was employed to determine the concentration of NOx.

### 2.8 Histology and immunohistochemistry

After fixation and demineralization, maxillae were paraffin‐embedded and sectioned. For histology, haematoxylin and eosin (HE) staining, tartrate‐resistant acid phosphatase (TRAP) staining and Masson's trichrome (Sigma‐Aldrich) staining were performed. For immunohistochemical analysis, Anti‐F4/80, anti‐ki‐67 and a fluorescent terminal deoxynucleotidyl transferase dUTP nick end labelling (TUNEL) apoptosis detection kit (Roche) was used to identify monocytes and macrophages.

### 2.9 ELISA and plasma biochemical assay

Plasma was collected when euthanizing animals. The tissue around the tooth sockets were obtained and homogenized to collect the supernatant. Mouse tartrate resistant acid phosphatase 5b (TRACPHOSPHORYLATED 5b) ELISA Kit (Elabscience) and carboxy‐terminal telopeptide of type‐I collagen (CTX‐1) ELISA Kit (J&L biological) was used to measure osteoclast activity according to the manufacturer's instructions. Mouse Nitric Oxide (NO) ELISA Kit (SAB) was used to measure the plasma NO level. Inducible Nitric Oxide Synthase (iNOS) Assay Kit (Beyotime) was used to measure the activity of iNOS. Alkaline Phosphatase (ALP), calcium ion (Ca^2+^) and phosphorus (P) levels in plasma were detected by automatic biochemical analyser to tests for osteogenesis.

### 2.10 Cytokines detection of tooth extraction sockets

The multiple cytokine levels of sockets were measured using the LEGENDplex mouse inflammation panel (740446, BioLegend, USA). According to standard experimental procedures provided by the manufacturer, tissues were separated and weighed for homogenization, and then diluted in a 96‐well plate with Assay Buffer. Tissue homogenates were centrifuged at 12,000 rpm for 10 min to collect the supernatant from tooth sockets tissue. 25 μl mixed beads were added to each well and incubated for 2 h at room temperature. After washing three times with 1 × Wash Buffer, 25 μl of Detection Antibodies was added to each well and incubated for 1 h. After that, 25 μl SA‐PE was added to each well and incubated for 30 min. Then the prepared samples were tested using FACS Symphony (BD LSRFortessa), and the data were analysed by LEGEND plex software v8.0 (Biolegend).

### 2.11 Flow cytometry analysis of intracellular NO levels and prodium iodide (PI)‐labelled monocyte

Monocytes were collected and stimulated as described as above. After 24 h, cells were collected, and incubated with intracellular NO probe (Beyotime), PI (Beyotime), respectively. All samples were acquired on a FACS LSRFortessa flow cytometer (BD Biosciences), and data were analysed by FlowJo software.

### 2.12 Cell viability assay

The cell viability was examined using a 3‐(4,5‐DimethylthiaZA‐2‐yl)‐2,5‐diphenyltetrazolium bromide (MTT) assay (Beyotime), which determined a formazan product due to the viable mitochondria in active cells. The media with 0.2% MTT solution were added and incubated at 37°C for 4 h. Cellular reduction of the MTT tetrazolium ring resulted in the formation of a purple water‐insoluble deposit of formazan crystals. The medium was discarded, and the reaction was stopped with dimethylsulfoxide and glycine buffer. The coloured product was transferred to a 96‐well plate and analysed by measuring the absorbance at 620 nm (A620).

### 2.13 Western blot analysis

Protein was extracted from the cells using radioimmunoprecipitation buffer and then measured the protein concentration using the bicinchoninic acid Protein Assay Kit. Briefly, protein fractions were separated using sodium dodecyl sulphate‐polyacrylamide gel electrophoresis and transferred to a polyvinylidene fluoride membrane. The membranes were blocked in a 5% non‐fat milk solution for 1 h at 25°C and incubated overnight at 4°C with the following primary antibodies: Phosphorylated RIPK3 Rabbit pAb (1:1000 dilution, AP1260, ABclonal, China), Phosphorylated MLKL‐T357/S358/S360 Rabbit pAb (1:1000 dilution, AP0949, ABclonal, China), MLKL Rabbit mAb (1:1000 dilution, A19685, ABclonal, China) and GAPDH (1:2000 dilution, 5174 T, Cell Signalling Technology, USA). After washing with Tris‐buffered saline, membranes were incubated with secondary antibodies for 1 h at room temperature. Bands were detected by chemiluminescence according to the manufacturer's instructions using a Bio‐Rad imaging system.

### 2.14 Statistical analyses

All statistical analyses were performed using GraphPad Prism 9 or the R (http://www.R-project.org). Unpaired Student's *t* test or one‐way ANOVA followed by Tukey's multiple comparison test was to evaluate differences between groups. Data are expressed as mean ± SD. Statistical analyses and graphical representation of data were carried out with GraphPad Prism 7.0. *p* < 0.05 was considered to be statistically significant.

## 3 RESULTS

### 3.1 Dietary nitrate improved tooth extraction sockets osseous wound healing after zoledronate administration in mice

**FIGURE 1:**
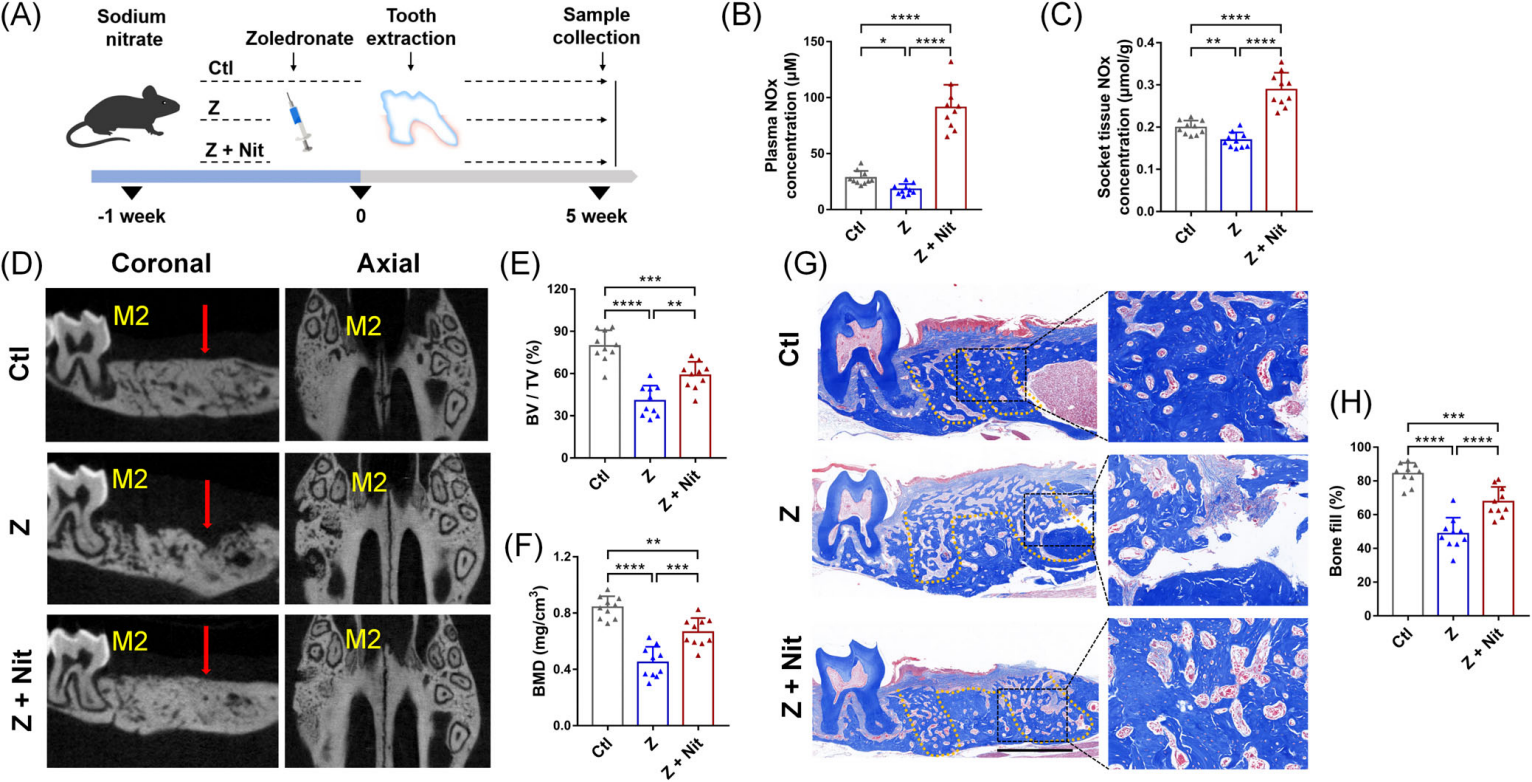
Effects of zoledronate therapy and nitrate administration on osseous healing at 5 weeks post‐extraction. (A) Schematic overview of study procedures: 4 mM nitrate was added to the drinking water of C57/BL6 mice 1 week before injection of zoledronate up to 5 weeks after extraction of the right upper first molar (Z + Nit). In the negative control group, there was no injection of zoledronate; only teeth were extracted (Ctl). In the positive control group, tooth were extracted after injection of zoledronate (Z). Mice were sacrificed 5 weeks after tooth extraction, and plasma and maxilla were harvested. (B) NOx concentration in plasma and (C) the NOx level of tooth extraction sockets and surrounding tissues in groups 5 weeks after tooth extraction. (D) Representative micro‐CT images (Red arrow: first molar mesial buccal root; M2: second molar). (E) The relative bone volume (BV/TV) and (F) bone mineral density (BMD) of the first molar mesial buccal root were measured. (G) Trichrome‐stained images of extraction sockets (yellow line: roots of tooth extraction sockets; bar = 1 mm) and (H) the area of blue‐stained bone tissue in sockets was quantified. *n* = 10/group. Data are presented as mean ± SD. *p < 0.05, **p < 0.01, ***p < 0.001, ****p < 0.0001, ns, not significant.

**FIGURE 2:**
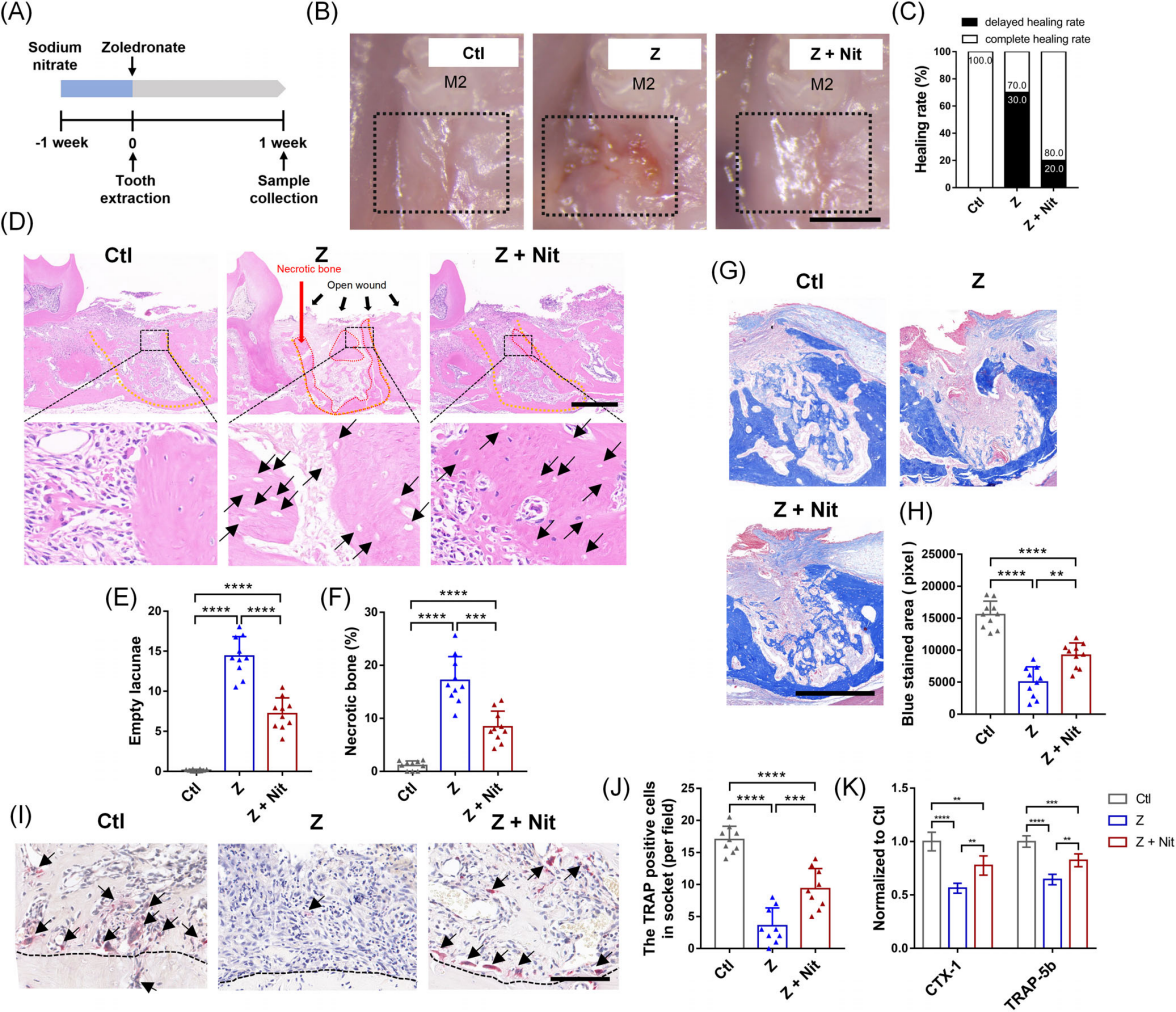
Effects of zoledronate therapy and nitrate administration on osseous healing 1 week post‐extraction. (A) Schematic overview of study procedures: 4 mM nitrate was added to the drinking water of C57/BL6 mice 1 week before injection of zoledronate up to 1 week after extraction of the right upper first molar (Z + Nit). In the negative control group, there was no injection of zoledronate; only the teeth were extracted (Ctl). In the positive control group, tooth were extracted after zoledronate injection (Z). Mice were sacrificed 1 weeks after extraction, and plasma and maxilla were harvested. (B) Appearance of mucosal healing at the extraction sites and (C) the incidence rate of the delayed healing socket post‐zoledronate and nitrate administration (black line: wounds, M2: second molar, bar = 2 mm). (D) Representative HE‐stained images of tooth extraction sockets of roots (yellow line: tooth extraction sockets; areas surrounded by red line: necrotic bone with empty lacunae; bar = 500 μm). (E), The number of osteocyte lacunae without nuclear stain were quantified as empty lacunae. (F) Areas containing 10 empty lacunae were quantified as necrotic and are presented as a percentage of the total bone present. (G) Representative trichrome‐stained images of tooth extraction sockets (bar = 500 μm) and (H) the blue‐stained bone trabeculae were quantified. (I, J) Representative TRAP‐stained images (black line: tooth extraction sockets; bar = 100 μm). The number of osteoclasts was significantly increased in the Z + Nit group versus the Z group. (K) Relative contents of CTX‐1 and Trap‐5b in the homogenate of extracted tooth sockets and surrounding tissue (normalized to Ctl group). *n* = 10/group. Data are mean ± SD. *p < 0.05, **p < 0.01, ***p < 0.001, ****p < 0.0001, ns, not significant.

To investigate the effects of dietary nitrate on jawbone remodelling in zoledronate‐treated mice, the BRONJ model was established, as shown in Figure 1A. There was no significant difference in water intake between the groups (Figure S1). The supplement of 4 mM nitrate in the drinking water significantly increased the NOx content in plasma (Figure 1B) and the socket surrounding tissues (Figure 1C). Analysis was carried out after dividing the regions of interest (ROIs) of the tissue (Figure S2). MicroCT showed that the alveolar bone of the Ctl group was healing at 3 weeks after tooth extraction, but there was no significant difference in bone mineral density between the alveolar tissue and the surrounding tissue at 5 weeks. So we chose 5 weeks after extraction as the long‐term observation point (Figure S3). MicroCT analysis (Figure 1D) revealed that 5 weeks after tooth extraction, Z + Nit group had significantly improved bone healing than that of Z group, which was confirmed by the quantitative analysis of bone mineral density (BMD) and relative bone volume (BV/TV) (Figure 2E, F). Histopathological staining indicated that the bone fill (%) in tooth socket in the Z + Nit group was better than that of in the Z group, showing more blue stained hard tissues in Figure 1G, H.

Next, we explored how inorganic nitrate promoted bone healing in the early stages. The BRONJ model was established, and dosing ended after tooth extraction (Figure 2A). The sockets in the Z group did not heal and showed incomplete epithelial coverage. In contrast, fewer delayed healing rate observed in the Z + Nit and Ctl groups (Figure 2B, C). Examples of complete healing and delayed healing are seen in Figure S4.

To further observe bone remodelling after tooth extraction, the slides of maxillary bone were HE‐stained and trichrome‐stained. Dietary nitrate significantly decreased in the necrotic bone (%) and empty lacunae area in the Z + Nit group compared with that in the Z group (Figure 2D–F), with a significantly increased blue‐stained trabecular area in the sockets (Figure 2G, H). TRAP staining and the levels of CTX‐1 and TRAPHOSPHORYLATED 5b were an important representation of the function and activity of osteoclasts. The TRAP staining showed that dietary nitrate rescued the zoledronate‐induced reduction of osteoclasts on bone surfaces in the sockets (Figure 2I, J). Significantly higher levels of CTX‐1 and TRAPHOSPHORYLATED 5b in the tissues surrounding the sockets in the Z + Nit group were also detected by ELISA (Figure 2K).

In addition, bone metabolism markers in plasma were also investigated, and a significantly higher level of alkaline phosphatase (ALP) was detected in the Z + Nit group compared with that in the other two groups. However, no significant difference in phosphorus or calcium ion content was observed in the plasma (Figure S5). The overall results indicated that dietary nitrate balanced the dynamics of osteogenesis and osteoclasts.

### 3.2 Dietary nitrate regulated the immune microenvironment of tooth extraction sockets and the surrounding tissues

**FIGURE 3:**
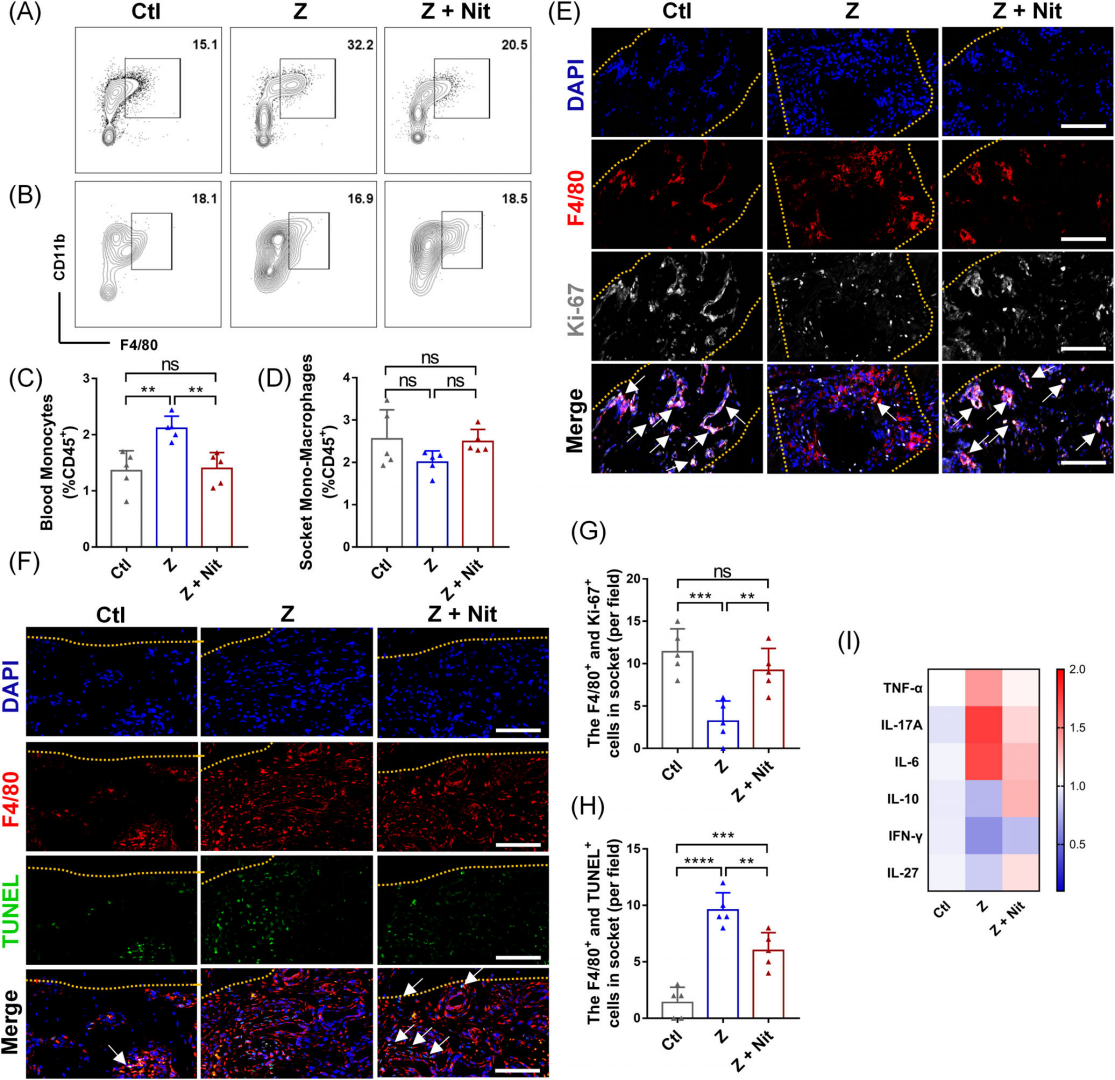
Effects of zoledronate therapy and nitrate administration on compromised soft tissue healing 1 week post‐extraction. (A) Flow cytometry examination of CD45^+^CD11b^+^CD11c^+/−^F4/80^+^Ly6C^+^ monocytes from mice in CD45^+^ peripheral blood cells. (B), Flow cytometry examination of CD45^+^CD11b^+^CD11c^+/−^F4/80^+^Ly6C^+^ monocytes from tooth sockets of mice and the surrounding tissue. (C) The summarized frequency of monocytes in CD45^+^ immune cells from peripheral blood cells. (D) The summarized frequency of monocytes in CD45^+^ immune cells from extracted tooth sockets and surrounding tissue. (E) Representative F4/80 and Ki‐67 immunofluorescent staining images of tooth extraction sockets (yellow line: tooth extraction sockets; blue fluorescence: DAPI^+^ cell nucleus; red fluorescence: F4/80^+^ Monocyte‐macrophages; white fluorescence: Ki‐67^+^ cells; pink fluorescence: F4/80^+^ Ki‐67^+^ dual‐positive cells represented the proliferating osteoclasts; bar = 100 μm). (F) Representative F4/80 and TUNEL immunofluorescent staining images of tooth extraction sockets (yellow line: tooth extraction sockets; blue fluorescence: DAPI^+^ cell nucleus; red fluorescence: F4/80^+^ monocyte‐macrophages; green fluorescence: TUNEL^+^ cells; green staining of the nucleus and red staining of the membrane represent monocyte‐macrophages in the process of death; bar = 100 μm). (G) The numbers of F4/80^+^ and Ki‐67^+^ dual‐positive osteoclasts per field. (H) The numbers of green staining of the nucleus and red staining of the membrane monocytes per field. (I) Relative contents of IL‐10, IFN‐γ, IL‐27 TNF‐α, IL‐17A and IL‐6 in the homogenate of extracted tooth sockets and surrounding tissue (normalized to the Ctl group). *n* = 5/group. Data are mean ± SD. *p < 0.05, **p < 0.01, ***p < 0.001, ****p < 0.0001, ns, not significant.

Since the monocytes played an important role in initiating and maintaining BRONJ,^25^ we investigated the effects of dietary nitrate on the monocytes and their related immune response in the soft tissue of tooth extraction sockets and the surrounding tissues for 1 week after tooth extraction. The frequency of mature monocytes in the soft tissue of socket and peripheral blood was determined by flow cytometry to evaluate the efficiency of monocytes exerted by zoledronate and nitrate (Figure S6). As shown in Figure 3A, C, compared with the Ctl group, injection of zoledronate increased the frequency of mature monocytes in peripheral blood in the Z group. In contrast, the Z + Nit group showed a reduced frequency of monocytes. However, there was no significant difference in the frequency of monocytes between the groups in the tooth extraction sockets and surrounding tissues (Figure 3B, D).

In addition, the results showed that a few F4/80 positive cells in the Z group were Ki‐67 positive proliferating cells, and more of them were TUNEL‐positive cells, revealing that zoledronate inhibited the proliferation and differentiation potential of monocytes, resulting in monocyte death. Nitrate significantly improved the survival and differentiation ratio of monocyte–macrophages compared with the Z group (Figure 3E–H).

Furthermore, dietary nitrate effectively reduced the levels of TNF‐α, IL‐17A and IL‐6 in the sockets and surrounding tissues and increased the levels of IL‐10, IFN‐γ and IL‐27 that were reduced by zoledronate (Figure 3I). Taken together, this data suggests that dietary nitrate could protect monocytes and regulate the immune microenvironment of sockets in the early stage of tooth extraction.

### 3.3 Dietary nitrate induced a distinct metabolomic pattern of monocyte

**FIGURE 4:**
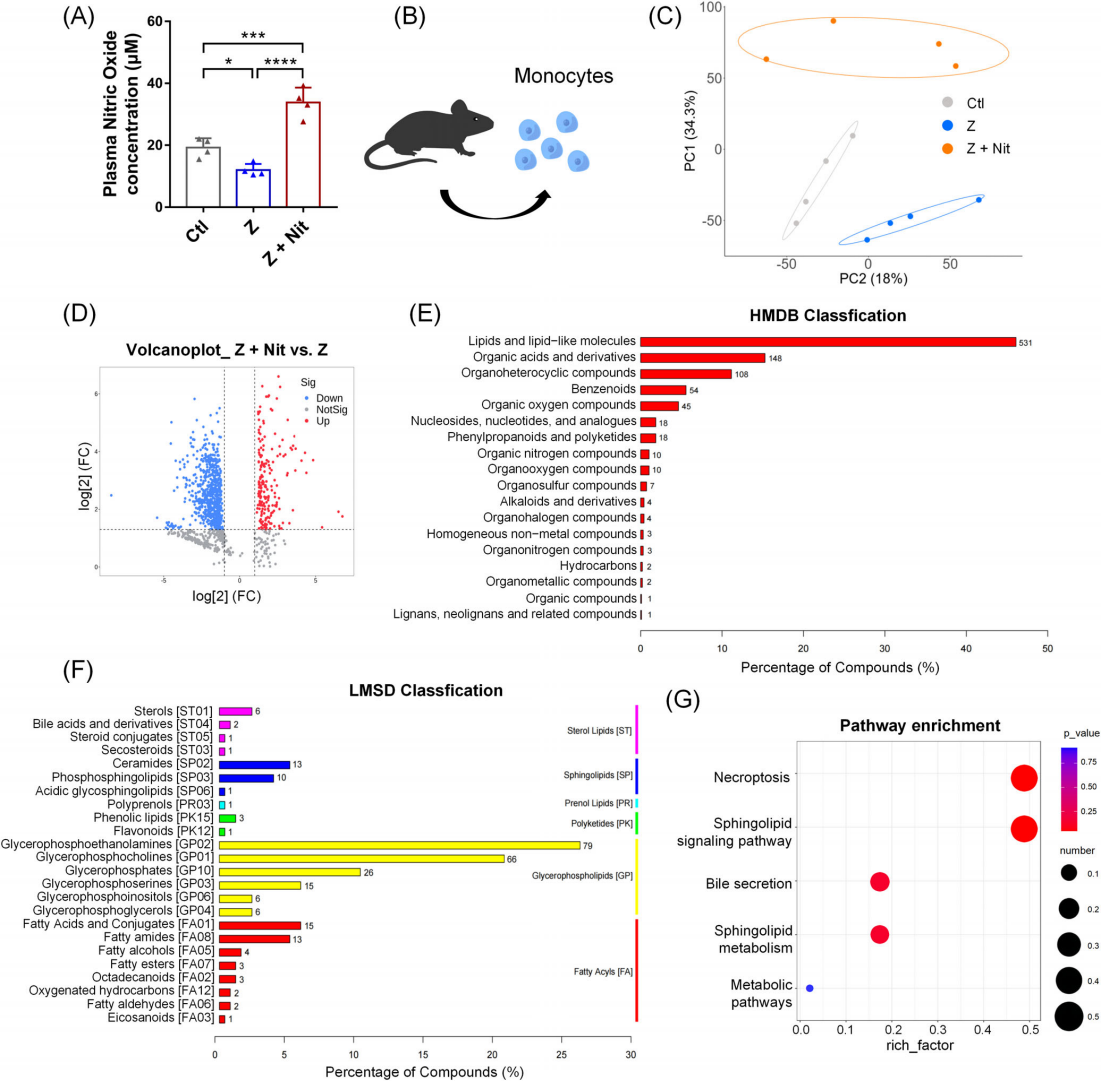
Non‐targeted metabolomics profiling analysis of monocytes from Ctl, Z and Z + Nit groups. (A) NO concentration in plasma. (B) Monocytes were isolated from mice. (C) PCA scores plot of Ctl, Z and Z + Nit groups. (D) Volcano plot showing differential metabolites between Z + Nit and Z groups. Red dots indicate differentially expressed and up‐regulated metabolites with corrected *p* < 0.05. Blue dots indicate differentially expressed and down‐regulated metabolites with corrected *p* < 0.05. (E) Human Metabolome Database classification (HMDB) analysis of differential metabolites between groups. (F) Lipid Maps Structure Database (LMSD) classification analysis of differential metabolites between groups. (G) Pathway enrichment analysis of differential metabolites that were used for visualization. *n* = 4/group. Data are mean ± SD. *p < 0.05, ***p < 0.001, ****p < 0.0001, ns, not significant.

After zoledronate injection, monocyte function could be affected and regulated by intracellular metabolism, resulting in a cytokine storm.^25^, ^26^ Since NO has a very powerful immune‐regulating function,^5^ and our results showed that 4 mM nitrate in drinking water could significantly increase the plasma NO content (Figure 4A). Then, we explored the metabolomic signature of monocytes in mice with or without dietary nitrate treatment (Figure 4B). Differentially expressed metabolites (DEMs) were identified using LC–MS. Levels of lipids and lipid‐like molecules were significantly different between the Ctl and Z groups (Table S1). The PCA scoring plot showed distinct separation in the Ctl, Z and Z + Nit groups (Figure 4C).

DEMs had an average ratio‐fold change >2, phosphorylated value <0.05 and variable importance in the projection (VIP) > 1. A set of 889 metabolites were screened as significantly changed in the Z + Nit group compared with the Z group, including 208 increased metabolites and 681 decreased metabolites (Figure 4D).

By using the Human Metabolome Database for classification, a large proportion of lipids and lipid‐like molecules were found to be significantly different in the Z + Nit group than in the Z group (Figure 4E). The Lipid Maps Structure Database classification for further biological interpretation was subsequently performed to reveal the most relevant lipid classification (Figure 4F). The non‐targeted metabolomic analysis showed significant perturbations in glycerophospholipids, fatty acids and sphingolipids (Figure S7). Next, pathway enrichment analysis was conducted using the KEGG database to reveal the potential biological functions of the metabolites (Figure 4G), the most significantly enriched pathway of the Z + Nit versus Z group was the necroptosis pathway. These pathways and metabolic regulations work together to change monocyte function, thus altering the surrounding immune environment.

### 3.4 NO pretreatment exerted a protective effect on monocytes induced by zoledronate in vitro

**FIGURE 5:**
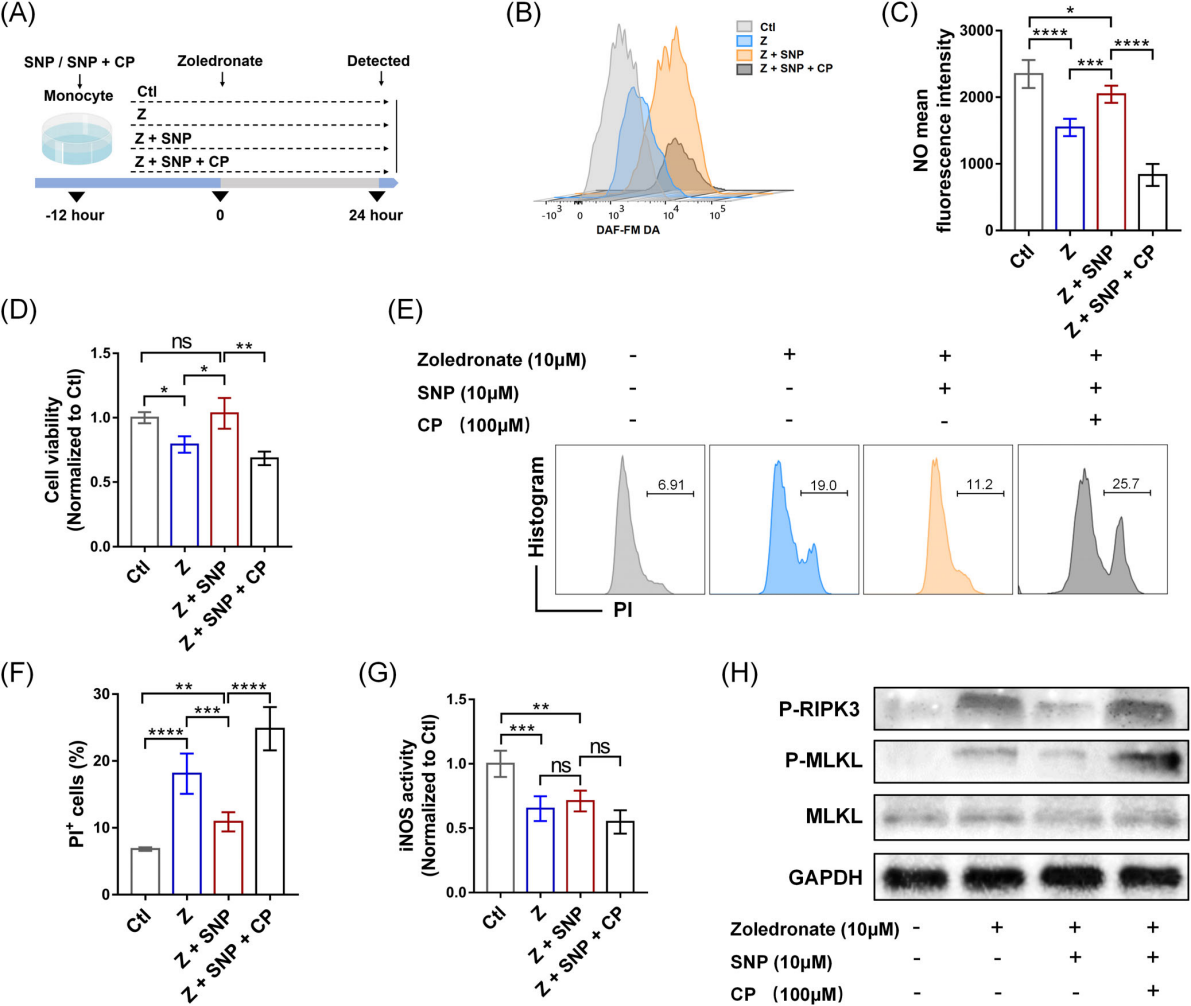
Nitric oxide (NO) is involved in monocyte regulation under zoledronate treatment in vitro. (A) Schematic overview of study procedures: 10 μM sodium nitroprusside (SNP) or 10 μM SNP + 100 μM Carboxy‐PTIO (CP) was added to the culture medium and incubated overnight. Monocytes were tested 24 h after the addition of 10 μM zoledronate. (B) Representative flow cytometric results of the NO level in groups at 24 h after zoledronate was added. (C) The relative mean fluorescence intensity of NO was quantified. (D) Cell viability of monocytes was measured by MTT assay. (E) Representative FACS plots and (F) the frequency of PI‐positive cells among monocyte. (G) The level of inducible nitric oxide synthase (iNOS) activity was detected. (H) The expressions of necroptosis‐related protein after zoledronate pretreatment for 24 h. *n* = 5/group. Data are mean ± SD. *p < 0.05, **p < 0.01, ***p < 0.001, ****p < 0.0001, ns, not significant.

Although the non‐targeted metabolomic analysis revealed significant metabolic differences between the groups, the pathogenesis of the alteration in lipids and lipid‐like molecules of monocytes induced by zoledronate and NO pretreatment is unknown. To investigate how zoledronate could influence monocyte viability, monocytes were isolated from C57/B6 mice and treated with 10 μM zoledronate. SNP ‐ a common NO donor, provided exogenous NO in the culture. An NO scavenger CP was also used in this study to block NO in monocytes.^27^ As shown in Figure 5A, monocytes (Figure S8A) were pretreated with 10 μM SNP or SNP + CP for 12 h and followed by 24 h Z stimulation. DAF‐FM DA was used to detect intracellular NO levels. NO levels were significantly reduced after 24 h zoledronate stimulation. Meanwhile, pretreatment with the NO donor effectively increased intracellular NO levels (Figure 5B, C).

**FIGURE 6:**
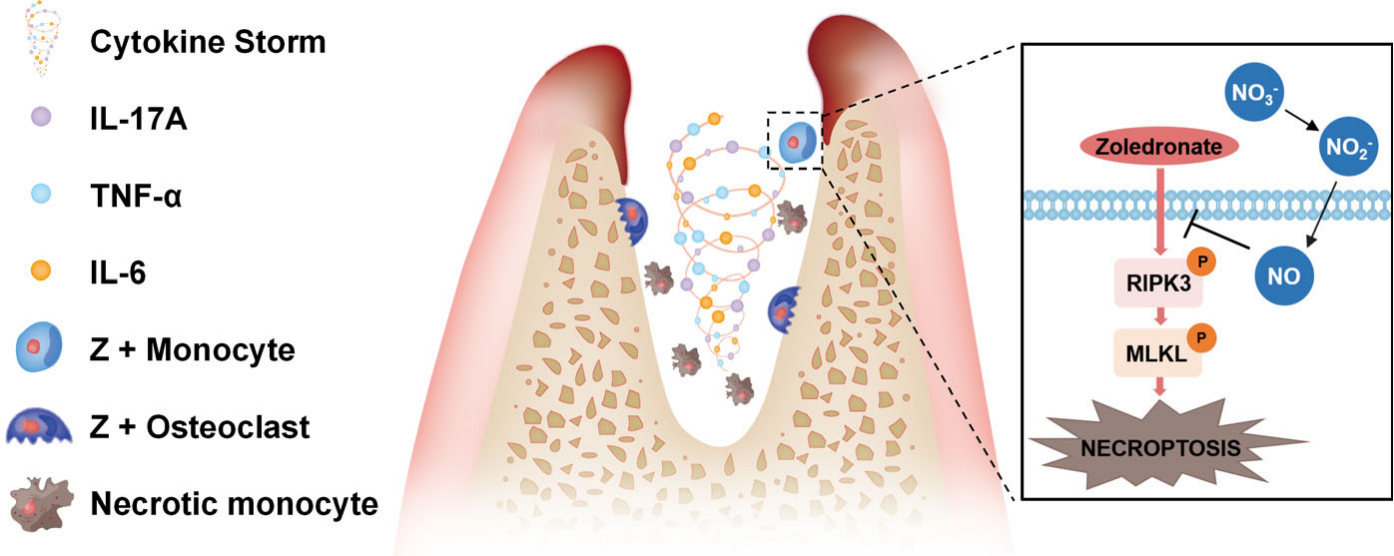
Proposed model: dietary nitrate could reduce monocyte necrosis stimulated by zoledronate by producing NO in vivo, thus regulating immunity and promoting bone remodelling after injury.

The protective effect of NO on monocytes was confirmed by measuring cell viability using the MTT assay (Figures 5D and S8B, C). Quantification by flow cytometry also showed that the Z + SNP group had a significantly reduced proportion of PI‐positive cells compared with the Z group (Figure 5E, F); however, the administration with CP could reverse the protection of SNP on monocytes. The iNOS activity of monocytes significantly decreased after Z was added (Figure 5G). We then further investigated the molecular mechanism underlying zoledronate‐induced toxicity in mouse monocytes and the protective effects of NO. Phosphorylated RIPK3 levels were significantly up‐regulated, along with related downstream signalling effectors, phosphorylated MLKL (Figure 5H). These results suggested that the necroptosis pathway was activated 24 h after zoledronate stimulation. Additionally, NO pretreatment attenuated the levels of necroptosis effectors, including phosphorylated RIPK3 and phosphorylated MLKL, to inhibit necroptosis. However, blocking NO in monocyte cells by CP could inhibit the alteration induced by SNP. Taken together, NO pretreatment regulates downstream metabolism by inhibiting the activity of phosphorylated RIPK3 and ultimately attenuates necrosis of monocytes induced by zoledronate (Figure 6).

The cells were also observed after the induction into osteoclasts. It was also found that NO can partially reverse the inhibition effect of zoledronate; the administration with CP could reverse the protective effect of SNP on monocytes (Figure S9).

## 4 DISCUSSIONS

This study demonstrated that dietary nitrate reduces BRONJ‐like lesions mainly by rescuing monocyte–macrophages in the tissue of tooth extraction sockets, suppressing inflammation, improving soft and bone tissue wound healing by promoting the production of bone trabecula.

The systemic effects of bisphosphonates have been extensively studied.^2^ Bisphosphonates accumulate in the bone matrix after a single injection,^3^ with an average half‐life of approximately 10 years.^28^ Further studies have shown that monocytes can ingest bisphosphonates in the blood. This affects normal monocyte metabolism such as farnesyl pyrophosphate synthase and intracellular accumulation of isopentenyl diphosphate pathway and dimethylallyl diphosphate, which accumulate intracellularly and are sensed by Vγ9/Vδ2T cells. After activation, Vγ9/Vδ2 T cells produce many proinflammatory cytokines, which affect the function of other cells in the microenvironment, including neutrophils and local tissue cells,^26^, ^29^ thus delaying wound healing. In addition, a single injection of zoledronate in the human body could increase the number of CD14^+^ monocytes in peripheral blood and increase the levels of TNF‐α and macrophage colony‐stimulating factor in plasma,^30^ leading to systemic proinflammatory effects in vivo.^25^

Previous studies have revealed the potential applications of NO in chronic wound healing. NO plays a complex regulatory role in the function, growth and apoptosis of various immune cells.^5^ However, in the application of NO gas therapy, the intrinsic shortcomings of gas therapy, such as short storage and release times, as well as the temporal and spatial uncontrollability of the release mode, should be resolved.^31^ Dietary nitrate is slowly and continuously converted to NO in response to oral and gastrointestinal bacteria. NO is transported through the blood to the body, where it plays an important role.^18^

In this study, dietary nitrate significantly decreased the open wound area of tooth extraction sockets, with alterations in bone parameters obtained from histomorphometry and micro‐CT analyses. The relationship between bone marrow stromal cells, osteoblasts and osteoclasts is a dynamic process in bone formation and remodelling. Excessive inhibition of osteoclasts and their precursors by monocytes can affect osteogenic activity in a dose‐dependent manner.^32^, ^33^ The recovery of osteoclasts by nitrate may contribute to bone remodelling. These processes involve the activation of bone turnover coupled with osteoblasts and osteoclasts. We found that zoledronate inhibited the proliferation and differentiation potential of monocytes, the precursors of osteoclasts, resulting in monocyte death in the tooth extraction sockets. However, injection of zoledronate increased the frequency of mature monocytes in peripheral blood. These findings suggest that BRONJ is not only a local lesion in the maxillary bone, but mechanically and more importantly, a systemic deterioration of the monocyte‐macrophage system caused by bisphosphonates.

Necroptosis is a non‐apoptotic form of regulated cell death that is dependent on RIPK3 and MLKL protein phosphorylation. Phosphorylated RIPK3 activiated the pore‐forming protein MLKL, facilitating its oligomerization and subsequent translocation to the plasma membrane, resulting in plasma membrane rupture and lipid molecule changes.^34^ Various classes of lipids in eukaryotic cells, such as glycerophospholipids,^35^ sphingolipids,^36^ and fatty acids,^37^ have been identified as downstream events for bioactive signalling molecules in the regulation of cell necroptosis. In addition to their classical roles in energy storage and as structural molecules, lipids have emerged as essential signalling regulators of inflammatory responses, differentiation, motility and cell death.^38^ As a result, unlike apoptosis, necroptosis could trigger or amplify inflammation and mediate a variety of inflammatory conditions,^39^ suggesting that it may be involved in BRONJ pathophysiology. In this study, zoledronate was ingested by monocytes, which are precursors of osteoclasts. In mice, dietary nitrate can be converted into NO, which can be transported to the body by the blood. Diffusion of NO through the cell membrane into monocytes can significantly inhibit necroptosis and regulate the expression of glycerophospholipid and sphingolipid metabolites, thereby affecting their functions, changing the release of cytokines in the surrounding environment, and regulating the function of bone cells after zoledronate injection.

The mechanisms of BRONJ is still not established and current hypotheses are complicated. Multiple factors, including monocytes, were reported to play a role in the initiation and pathogenesis of BRONJ. Several treatments have shown beneficial effects against BRONJ, such as stem cell transplantation,^40^ angiogenesis factor,^41^ extracellular vesicles.^42^ However, cost, uncertain efficacy, potential side effects, tolerance issues, immunogenicity and/or ethical issues have delayed the translation of these therapeutic strategies to the clinical setting. The short‐acting effect of organic nitrates, intolerance and side effects—such as headache, bradycardia, flushing, nausea and vomiting—limit the use of these organic nitrates. Inorganic nitrate is a hydrophilic salt, produced endogenously and exogenously. It does not undergo first‐pass metabolism by the liver and is readily absorbed by the intestinal mucosa. Furthermore, inorganic nitrate lacks immunogenicity, is not subject to tolerance, is non‐toxic and does not induce significant side effects at regular doses.^43^ Based on our previous studies,^12^, ^13^ 4 mM nitrate supplementation alone has no significant effect on the body without other challenging. Therefore, inorganic nitrate may have wide clinical applications.

In the present study, we found that the proliferation and differentiation potential of monocytes in tooth extraction was inhibited by zoledronate. In peripheral blood, however, the number of monocytes increased. To further investigate the effect of zoledronate on monocytes, we conducted in vitro experiments. With zoledronate stimulation, the NO levels in monocytes decreased significantly, while SNP, which releases NO, increased the viability and decreased the impairment of monocytes caused by zoledronate. Furthermore, with NO loading, the phosphorylated RIPK3 driven necroptosis pathway was inhibited, alleviating MLKL oligomerization, as verified by western blotting. To further investigate the effects of zoledronate on monocytes and the protection mechanisms of the nitrate/NO axis on BRONJ in vitro, CP was used in the cell culture system. Necroptosis could be activated in monocytes by zoledronate stimulation, while SNP loading could down‐regulate this effect. CP could reverse the protective effects of SNP on zoledronate‐treated monocytes. Our data suggested that NO, which was derived from nitrate, could protect monocytes from zoledronate‐induced necrosis.

In summary, our data revealed a novel regulatory mechanism of the metabolomic signature on innate immune responses induced by zoledronate. Furthermore, we found that dietary nitrate can regulate monocytes stimulated by zoledronate by producing NO in vivo, thus regulating immunity and promoting bone remodelling after injury. These data might contribute to the understanding of the immunopathogenesis of zoledronate and support the feasibility of dietary nitrate for the clinical prevention of BRONJ.

## AUTHOR CONTRIBUTIONS

Wen Pan and Jianyu Gu contributed to conception, design, data acquisition, analysis and interpretation, drafted the manuscript; Shihan Xu, Chunmei Zhang, Jinsong Wang contributed to data acquisition and performed all statistical analyses; Songlin Wang and Junji Xu contributed to conception, design, interpretation and critically revised the manuscript. All authors gave final approval and agree to be accountable for all aspects of the work.

## CONFLICT OF INTEREST

The authors declare no conflict of interest.

## Supporting information


**DATA S1.** Supporting Information

## Data Availability

All data associated with this study are present in the article itself or in the supplementary information file. All other relevant data are available from the authors upon reasonable request.

## References

[1] Marx RE . Pamidronate (Aredia) and zoledronate (Zometa) induced avascular necrosis of the jaws: a growing epidemic. J Oral Maxillofac Surg. 2003;61(9):1115‐1117. 12966493 10.1016/s0278-2391(03)00720-1

[2] Ruggiero SL , Dodson TB , Fantasia J , et al. American association of oral and maxillofacial surgeons position paper on medication‐related osteonecrosis of the jaw: 2014 update. J Oral Maxillofac Surg. 2014;72(10):1938‐1956. 25234529 10.1016/j.joms.2014.04.031

[3] Elsayed R , Abraham P , Awad ME , et al. Removal of matrix‐bound zoledronate prevents post‐extraction osteonecrosis of the jaw by rescuing osteoclast function. Bone. 2018;110:141‐149. 29408511 10.1016/j.bone.2018.01.030 PMC5878730

[4] Lundberg JO , Carlström M , Weitzberg E . Metabolic effects of dietary nitrate in health and disease. Cell Metab. 2018;28(1):9‐22. 29972800 10.1016/j.cmet.2018.06.007

[5] Bogdan C , Röllinghoff M , Diefenbach A . The role of nitric oxide in innate immunity. Immunol Rev. 2000;173:17‐26. 10719664 10.1034/j.1600-065x.2000.917307.x

[6] Kalyanaraman H , Ramdani G , Joshua J , et al. A novel, direct NO donor regulates osteoblast and osteoclast functions and increases bone mass in ovariectomized mice. J Bone Miner Res. 2017;32(1):46‐59. 27391172 10.1002/jbmr.2909 PMC5199609

[7] Kim SM , Yuen T , Iqbal J , Rubin MR , Zaidi M . The NO‐cGMP‐PKG pathway in skeletal remodeling. Ann N Y Acad Sci. 2021;1487(1):21‐30. 32860248 10.1111/nyas.14486 PMC7914295

[8] Silva RO , Lucetti LT , Wong DV , et al. Alendronate induces gastric damage by reducing nitric oxide synthase expression and NO/cGMP/K(ATP) signaling pathway. Nitric Oxide. 2014;40:22‐30. 24831353 10.1016/j.niox.2014.05.002

[9] Jung K , Kim J , Ahn G , et al. Alendronate alleviates the symptoms of experimental autoimmune encephalomyelitis. Int Immunopharmacol. 2020;84:106534. 32361191 10.1016/j.intimp.2020.106534

[10] Park JW , Piknova B , Huang PL , Noguchi CT , Schechter AN . Effect of blood nitrite and nitrate levels on murine platelet function. PLoS One. 2013;8(2):e55699. 23383344 10.1371/journal.pone.0055699 PMC3562242

[11] Feng X , Wu Z , Xu J , et al. Dietary nitrate supplementation prevents radiotherapy‐induced xerostomia. Elife. 2021;10:e70710. 34581269 10.7554/eLife.70710 PMC8563005

[12] Li S , Jin H , Sun G , et al. Dietary inorganic nitrate protects hepatic ischemia‐reperfusion injury through NRF2‐mediated antioxidative stress. Front Pharmacol. 2021;12(616):634115. 34163351 10.3389/fphar.2021.634115 PMC8215696

[13] Chang S , Hu L , Xu Y , et al. Inorganic nitrate alleviates total body irradiation‐induced systemic damage by decreasing reactive oxygen species levels. Int J Radiat Oncol Biol Phys. 2019;103(4):945‐957. 30458235 10.1016/j.ijrobp.2018.11.021

[14] Kapil V , Khambata RS , Jones DA , et al. The noncanonical pathway for In vivo nitric oxide generation: the nitrate‐nitrite‐nitric oxide pathway. Pharmacol Rev. 2020;72(3):692‐766. 32576603 10.1124/pr.120.019240

[15] Hokugo A , Kanayama K , Sun S , et al. Rescue bisphosphonate treatment of alveolar bone improves extraction socket healing and reduces osteonecrosis in zoledronate‐treated mice. Bone. 2019;123:115‐128. 30926440 10.1016/j.bone.2019.03.027 PMC7282713

[16] Bi Y , Gao Y , Ehirchiou D , et al. Bisphosphonates cause osteonecrosis of the jaw‐like disease in mice. Am J Pathol. 2010;177(1):280‐290. 20472893 10.2353/ajpath.2010.090592 PMC2893671

[17] Yan R , Jiang R , Hu L , Deng Y , Wen J , Jiang X . Establishment and assessment of rodent models of medication‐related osteonecrosis of the jaw (MRONJ). Int J Oral Sci. 2022;14(1):41. 35948539 10.1038/s41368-022-00182-4 PMC9365764

[18] Lizheng Qin SW . Protective roles of inorganic nitrate in health and diseases. Curr Med. 2022;1(1):4.

[19] Patntirapong S , Poolgesorn M . Alteration of macrophage viability, differentiation, and function by bisphosphonates. Oral Dis. 2018;24(7):1294‐1302. 29869362 10.1111/odi.12908

[20] Bedogni A , Blandamura S , Lokmic Z , et al. Bisphosphonate‐associated jawbone osteonecrosis: a correlation between imaging techniques and histopathology. Oral Surg Oral Med Oral Pathol Oral Radiol Endod. 2008;105(3):358‐364. 18280968 10.1016/j.tripleo.2007.08.040

[21] Cho YA , Yoon HJ , Lee JI , Hong SP , Hong SD . Histopathological features of bisphosphonate‐associated osteonecrosis: findings in patients treated with partial mandibulectomies. Oral Surg Oral Med Oral Pathol Oral Radiol. 2012;114(6):785‐791. 23159117 10.1016/j.oooo.2012.08.457

[22] Yamashita J , Koi K , Yang DY , McCauley LK . Effect of zoledronate on oral wound healing in rats. Clin Cancer Res. 2011;17(6):1405‐1414. 21149614 10.1158/1078-0432.CCR-10-1614 PMC3060285

[23] Weitzberg E , Lundberg JO . Novel aspects of dietary nitrate and human health. Annu Rev Nutr. 2013;33:129‐159. 23642194 10.1146/annurev-nutr-071812-161159

[24] Basaqr R , Skleres M , Jayswal R , Thomas DT . The effect of dietary nitrate and vitamin C on endothelial function, oxidative stress and blood lipids in untreated hypercholesterolemic subjects: a randomized double‐blind crossover study. Clin Nutr. 2021;40(4):1851‐1860. 33115598 10.1016/j.clnu.2020.10.012

[25] Zhu W , Xu R , Du J , et al. Zoledronic acid promotes TLR‐4‐mediated M1 macrophage polarization in bisphosphonate‐related osteonecrosis of the jaw. FASEB J. 2019;33(4):5208‐5219. 30624969 10.1096/fj.201801791RR

[26] Munoz MA , Fletcher EK , Skinner OP , et al. Bisphosphonate drugs have actions in the lung and inhibit the mevalonate pathway in alveolar macrophages. Elife. 2021;10:10. 10.7554/eLife.72430 PMC8718110 34967731

[27] Liu L , Huang Z , Chen J , Wang J , Wang S . Protein phosphatase 2A activation mechanism contributes to JS‐K induced caspase‐dependent apoptosis in human hepatocellular carcinoma cells. J Exp Clin Cancer Res. 2018;37(1):142. 29986744 10.1186/s13046-018-0823-2 PMC6038275

[28] Papapoulos SE , Cremers SC . Prolonged bisphosphonate release after treatment in children. N Engl J Med. 2007;356(10):1075‐1076. 10.1056/NEJMc062792 17347467

[29] Roelofs AJ , Jauhiainen M , Mönkkönen H , Rogers MJ , Mönkkönen J , Thompson K . Peripheral blood monocytes are responsible for gammadelta T cell activation induced by zoledronic acid through accumulation of IPP/DMAPP. Br J Haematol. 2009;144(2):245‐250. 19016713 10.1111/j.1365-2141.2008.07435.x PMC2659391

[30] Raffray L , Burton RJ , Baker SE , Morgan MP , Eberl M . Zoledronate rescues immunosuppressed monocytes in sepsis patients. Immunology. 2020;159(1):88‐95. 31606902 10.1111/imm.13132 PMC6904622

[31] Wu M , Lu Z , Wu K , Nam C , Zhang L , Guo J . Recent advances in the development of nitric oxide‐releasing biomaterials and their application potentials in chronic wound healing. J Mater Chem B. 2021;9(35):7063‐7075. 34109343 10.1039/d1tb00847a

[32] Van Beek ER , Löwik CW , Papapoulos SE . Bisphosphonates suppress bone resorption by a direct effect on early osteoclast precursors without affecting the osteoclastogenic capacity of osteogenic cells: the role of protein geranylgeranylation in the action of nitrogen‐containing bisphosphonates on osteoclast precursors. Bone. 2002;30(1):64‐70. 11792566 10.1016/s8756-3282(01)00655-x

[33] Davison NL , Gamblin AL , Layrolle P , Yuan H , de Bruijn JD , Barrère‐de GF . Liposomal clodronate inhibition of osteoclastogenesis and osteoinduction by submicrostructured beta‐tricalcium phosphate. Biomaterials. 2014;35(19):5088‐5097. 24698521 10.1016/j.biomaterials.2014.03.013

[34] Galluzzi L , Vitale I , Aaronson SA , et al. Molecular mechanisms of cell death: recommendations of the nomenclature committee on cell death 2018. Cell Death Differ. 2018;25(3):486‐541. 29362479 10.1038/s41418-017-0012-4 PMC5864239

[35] Quarato G , Guy CS , Grace CR , et al. Sequential engagement of distinct MLKL phosphatidylinositol‐binding sites executes necroptosis. Mol Cell. 2016;61(4):589‐601. 26853145 10.1016/j.molcel.2016.01.011 PMC4769881

[36] Zhang X , Kitatani K , Toyoshima M , et al. Ceramide nanoliposomes as a MLKL‐dependent, necroptosis‐inducing, chemotherapeutic reagent in ovarian cancer. Mol Cancer Ther. 2018;17(1):50‐59. 29079707 10.1158/1535-7163.MCT-17-0173 PMC5752574

[37] Parisi LR , Li N , Atilla‐Gokcumen GE . Very long chain fatty acids are functionally involved in necroptosis. Cell Chem Biol. 2017;24(12):1445‐1454.e1448. 29033315 10.1016/j.chembiol.2017.08.026

[38] Hannun YA , Bell RM . Functions of sphingolipids and sphingolipid breakdown products in cellular regulation. Science. 1989;243(4890):500‐507. 2643164 10.1126/science.2643164

[39] Aguirre JI , Castillo EJ , Kimmel DB . Biologic and pathologic aspects of osteocytes in the setting of medication‐related osteonecrosis of the jaw (MRONJ). Bone. 2021;153:116168. 34487892 10.1016/j.bone.2021.116168 PMC8478908

[40] Li Y , Xu J , Mao L , et al. Allogeneic mesenchymal stem cell therapy for bisphosphonate‐related jaw osteonecrosis in Swine. Stem Cells Dev. 2013;22(14):2047‐2056. 23461552 10.1089/scd.2012.0615 PMC3699896

[41] Gao SY , Lin RB , Huang SH , et al. PDGF‐BB exhibited therapeutic effects on rat model of bisphosphonate‐related osteonecrosis of the jaw by enhancing angiogenesis and osteogenesis. Bone. 2021;144:115117. 31676407 10.1016/j.bone.2019.115117

[42] Watanabe J , Sakai K , Urata Y , Toyama N , Nakamichi E , Hibi H . Extracellular vesicles of stem cells to prevent BRONJ. J Dent Res. 2020;99(5):552‐560. 32119600 10.1177/0022034520906793

[43] Omar SA , Artime E , Webb AJ . A comparison of organic and inorganic nitrates/nitrites. Nitric Oxide. 2012;26(4):229‐240. 22491087 10.1016/j.niox.2012.03.008

