# Supplementary material for: Dietary nitrate improves jaw bone remodelling in zoledronate‐treated mice
Source: Cell Prolif. 2023 Feb 21;56(7):e13395. doi: 10.1111/cpr.13395 (PMC10334281; doi:10.1111/cpr.13395)
Supplement: Supplementary file 1 — DATA S1. Supporting Information [file CPR-56-e13395-s001.docx]

**Supporting Information**

**Dietary nitrate improves jaw bone remodeling in zoledronate-treated mice**

Wen Pan^1,2^, Jianyu Gu^1,2^, Shihan Xu^1,2^, Chunmei Zhang^1,2,3,4^, Jinsong Wang^1,2^, Songlin Wang^1,2,3,4,5^*, Junji Xu^1,3,4,5,6,7^*

^1^Salivary Gland Disease Center and Beijing Key Laboratory of Tooth Regeneration and Function Reconstruction, School of Stomatology, Beijing Laboratory of Oral Health, Capital Medical University, Beijing, China.

^2^Department of Biochemistry and Molecular Biology, School of Basic Medical Sciences, Capital Medical University, Beijing, China.

^3^Immunology Research Center for Oral and Systemic Health, Beijing Friendship Hospital, Capital Medical University, Beijing, China.

^4^Laboratory for Oral and General Health Integration and Translation, Beijing Tiantan Hospital, Capital Medical University, Beijing, China.

^5^Research Units of Tooth Development and Regeneration, Chinese Academy of Medical Sciences, Beijing, China

^6^Department of Periodontics, Beijing Stomatological Hospital, Capital Medical University School of Stomatology, Beijing, China.

^7^Shanghai Stomatological Hospital & School of Stomatology, Fudan University, Shanghai, China

Wen Pan and Jianyu Gu contributed equally to this work.

***Correspondence**:

Songlin Wang, Salivary Gland Disease Center and Beijing Key Laboratory of Tooth Regeneration and Function Reconstruction, School of Stomatology, Beijing Laboratory of Oral Health, Capital Medical University, Beijing, 100050, China. Email: slwang@ccmu.edu.cn.

Junji Xu, Department of Periodontics, Beijing Stomatological Hospital, Capital Medical University School of Stomatology, Tian Tan Xi Li No.4, Beijing 100050, China. Email: [uujkl@163.com](mailto:uujkl@163.com).

**Materials and methods**

**Osteoclast formation**

Bone marrow monocytes were cultured for 7 days in the presence of M-CSF (50 ng/mL) and RANKL (200 ng/mL) for differentiation into mature osteoclasts. Media were refreshed every 2 days.

**RNA extraction and quantitative real-time PCR**

Total RNA was isolated using the RNeasy mini kit (Qiagen). For reverse transcription, mRNA (500 ng) was reverse transcribed to cDNA using the RevertAid First Strand cDNA Synthesis Kit (Thermo Fisher Scientific). These steps were performed in accordance with the manufacturer’s instructions. cDNAs were stored at -20℃ or used immediately.

Quantitative real-time PCR samples were prepared using the SYBR GreenER qPCR

SuperMix Universal (Thermo Fisher Scientific). The amplification was carried out in a Step one plusTM Real-Time PCR system (Applied Biosystems; ABI). An initial denaturation step of 20 s at 95°C was followed by the appropriate amplification cycles.

The forward/reverse primer pairs were as follows:

| *GAPDH* | 5’ - GGAGCGAGATCCCTCCAAAAT - 3’ |
| --- | --- |
|  | 5’ - GGCTGTTGTCATACTTCTCATGG - 3’ |
| *CTSK* | 5’ - CTCGGCGTTTAATTTGGGAGA - 3’ |
|  | 5’ - TCGAGAGGGAGGTATTCTGAGT - 3’ |
| *ACP5* | 5’ - CACTCCCACCCTGAGATTTGT - 3’ |
|  | 5’ - CCCCAGAGACATGATGAAGTCA - 3’ |
| *NFATC1* | 5’ - GGAGAGTCCGAGAATCGAGAT - 3’ |
|  | 5’ - TTGCAGCTAGGAAGTACGTCT - 3’ |

For the analysis, the transcript copy numbers were normalized with that of glyceraldehyde 3-phosphate dehydrogenase-coding *GAPDH*, a relatively invariant housekeeping gene. The data were analyzed by the comparative CT method using StepOneTM Software version 2.2.2 (Applied Biosystems; ABI) and presented as fold changes relative to the untreated control mRNA level.


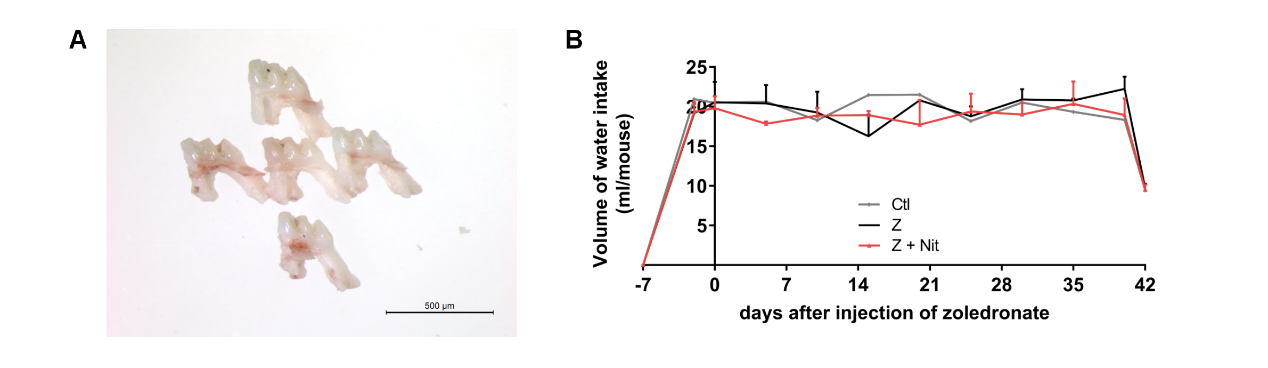


**Supplementary figure 1.** Drink intake in Ctl, Z and Z + Nit groups. n = 10/group.


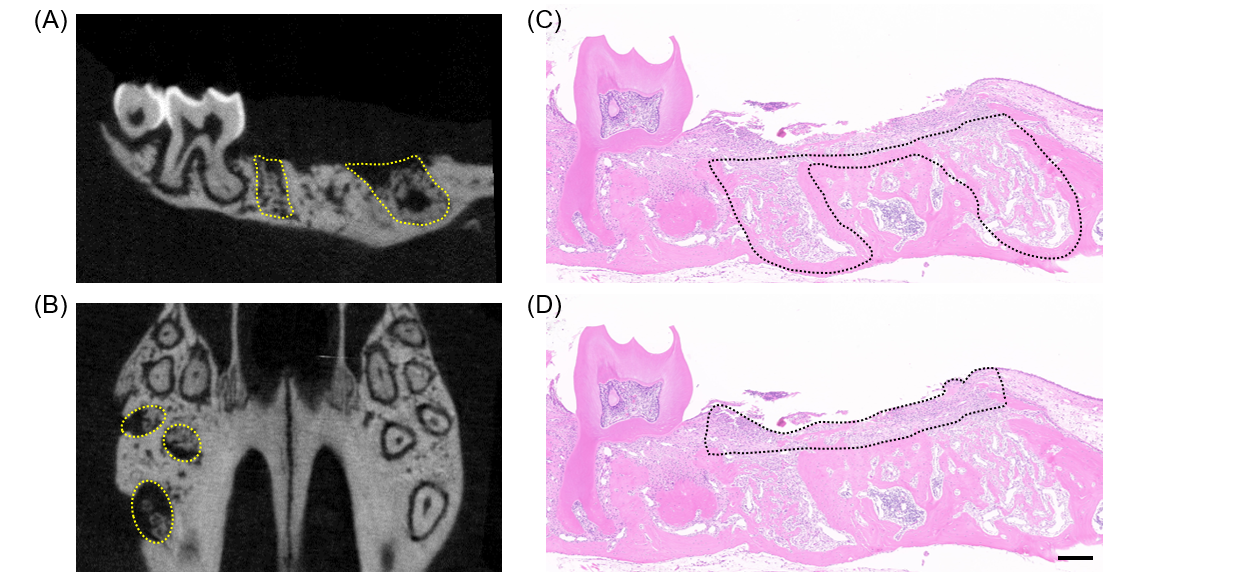


**Supplementary figure 2. A-B,** Regions of interest (ROIs) were drawn in the hard tissue of tooth extraction sockets. **C,** ROIs in the hard tissue of tooth extraction sockets (area surrounded by black dotted line). **D,** ROIs in the soft tissue of tooth extraction sockets (area surrounded by black dotted line, bar = 200μm).


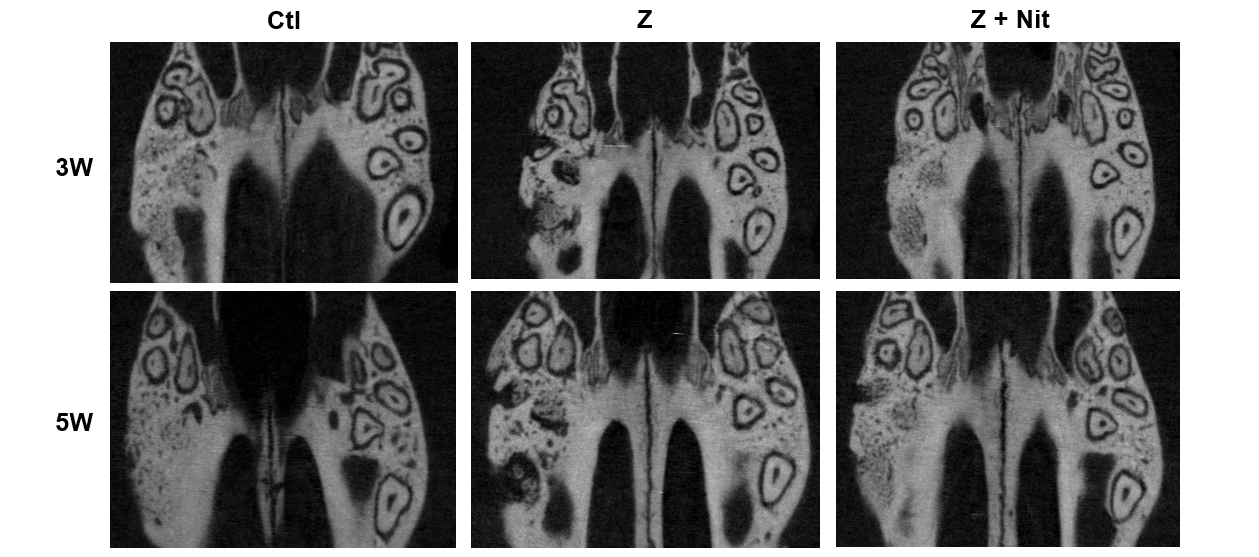


**Supplementary figure 3.** Representative micro-CT images at 3 weeks after extraction.


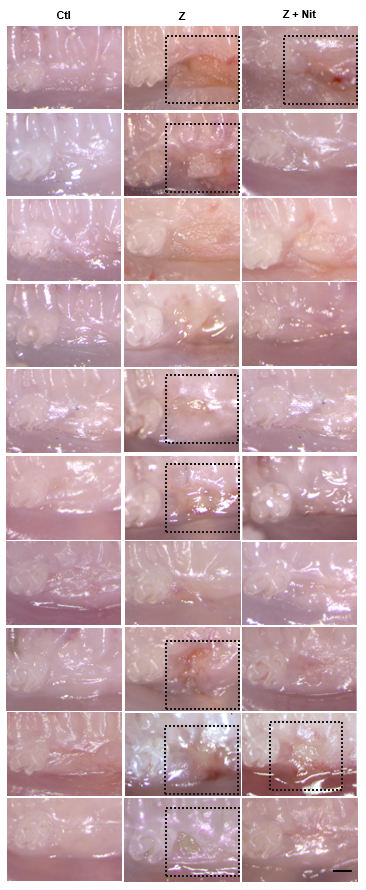


**Supplementary figure 4.** Comparison on occlusal photos of tooth extraction sockets in mice at one week after extraction (bar = 200μm). The tissue enclosed by the black dotted line is the area of delayed healing.


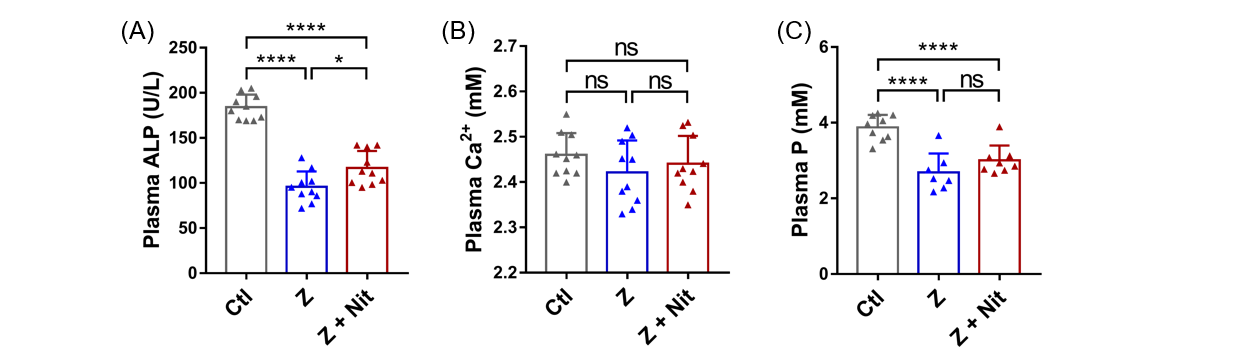


**Supplementary figure 5.** Monitoring plasma biochemical indices. **A,** Plasma levels of alkaline phosphatase (ALP). **B,** Plasma levels of calcium ion (Ca^2+^). **C,** Plasma levels of phosphorus (P). Data are mean ± SD. *P < 0.05, **P < 0.01, ****P < 0.0001, ns = not significant. n = 10/group.


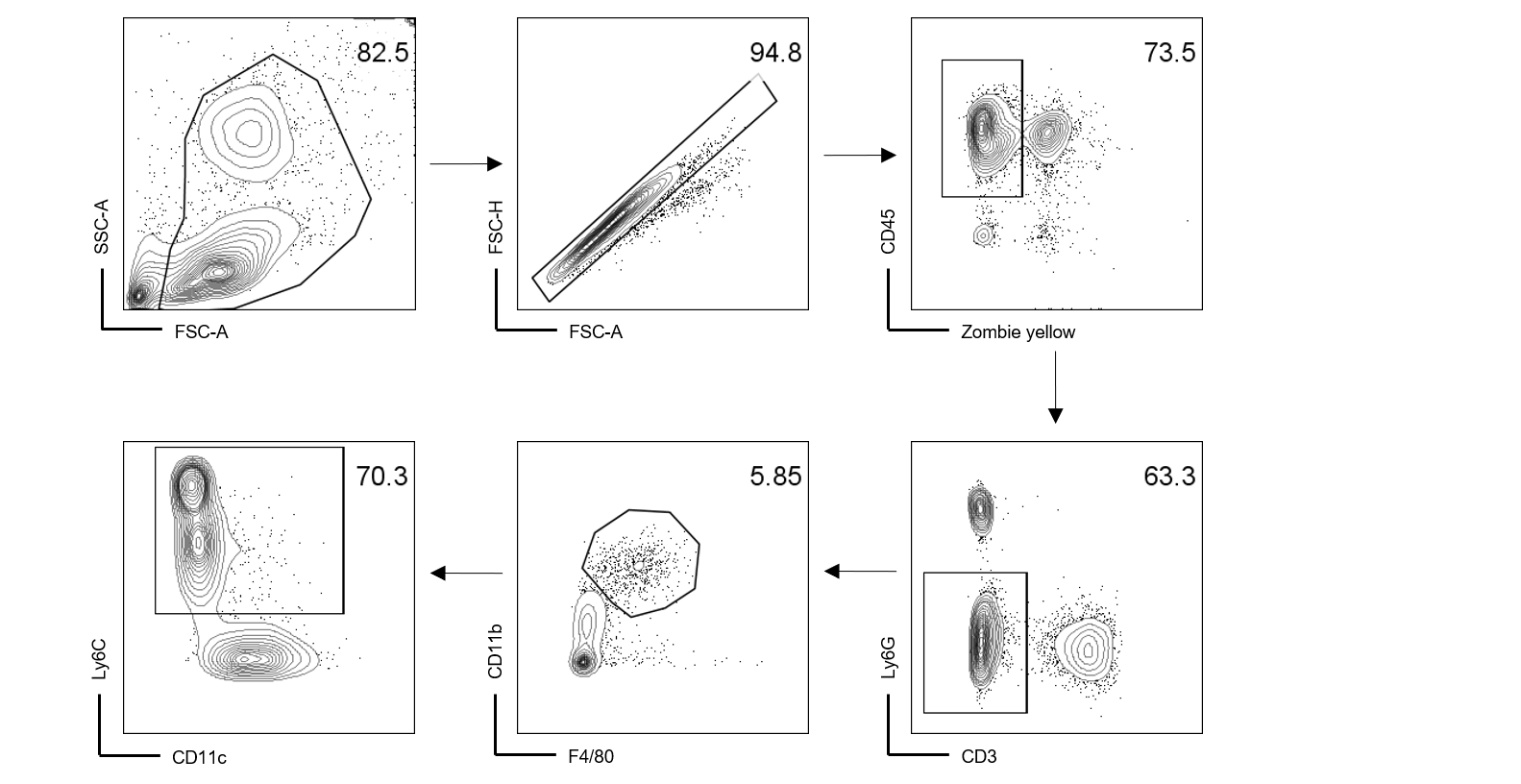


**Supplementary figure 6.** Representative plots for the gating strategy to identify monocytes of live CD45 ^+^CD11b^+^CD11c^+/-^F4/80^+^Ly6C^+^ (excludes CD3^+^Ly6G^+^) cells in the peripheral blood and tissues.


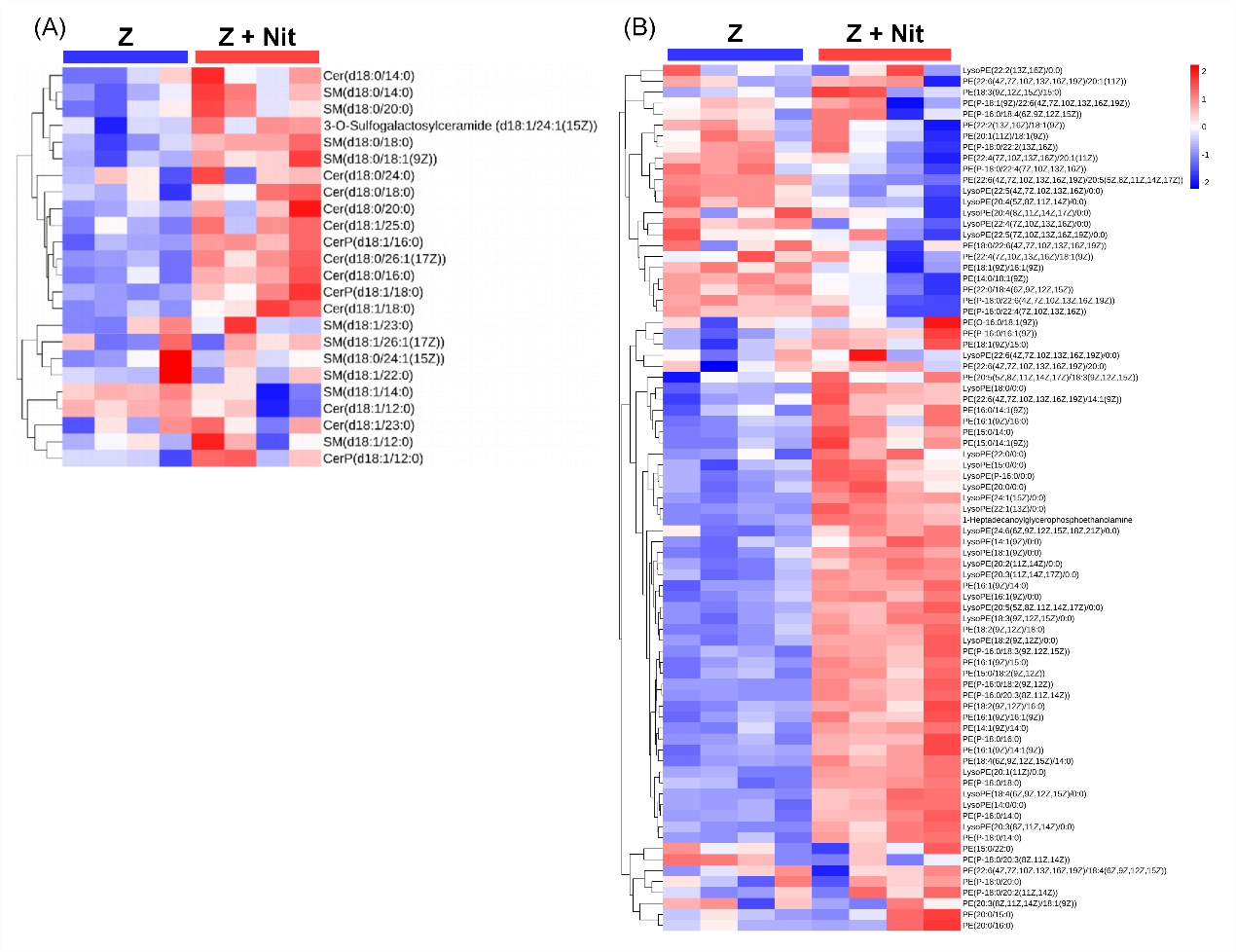


**Supplementary figure 7. A,** Heatmap representing the contents of 24 differential metabolites in sphingolipids. **B,** Heatmap representing the contents of 79 differential metabolites in glycerophospholipids, showing clear separation of Z + Nit and Z group.


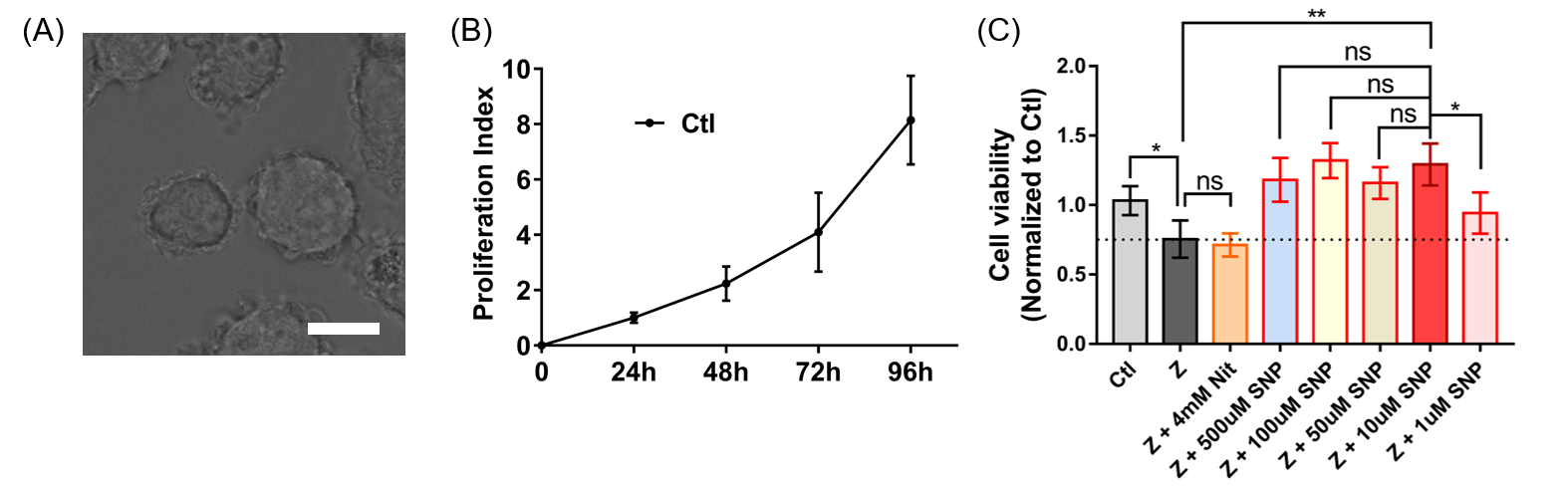


**Supplementary figure 8. A,** Morphology of monocytes. **B,** Proliferation curves of monocytes detected by MTT assay. **C,** Effects of different concentrations of SNP on monocyte viability detected by MTT assay. Bar = 10 μm, n = 5/group. Data are mean ± SD. *P < 0.05, **P < 0.01, ns = not significant.


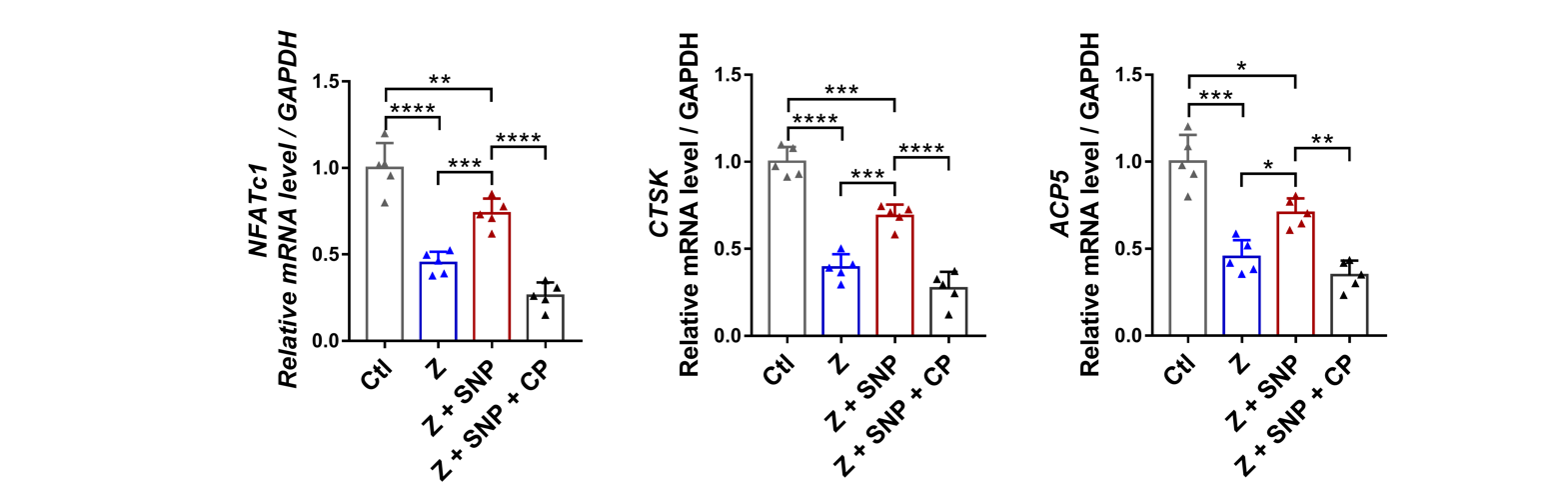


**Supplementary figure 9.** Relative mRNA levels of *NFATC1*, *CTSK*, and *ACP5* in monocytes differentiated toward osteoclasts. Data are mean ± SD. *P < 0.05, **P < 0.01, ***P < 0.001, ****P < 0.0001.

**Supplementary table 1.** Significantly altered monocytes metabolies in zoledronate-treated mice.

| Classification | Metabolite | VIP* | P-value† | FC‡ | Trend | AUC |
| --- | --- | --- | --- | --- | --- | --- |
| Benzenoids | Threo-Syringoylglycerol | 2.93 | 2.76E-02 | 0.30 | Down | 0.938 |
|  | 1'-Acetoxychavicol | 2.93 | 4.69E-03 | 0.27 | Down | 1.000 |
| Hydrocarbons | 3-Allyl-1-cyclohexene | 2.29 | 1.44E-02 | 0.35 | Down | 0.938 |
| Lipids and lipid-like molecules | 1,11-Undecanedicarboxylic acid | 1.66 | 3.96E-03 | 2.03 | Up | 1.000 |
|  | PE(P-18:1(9Z)/22:4(7Z,10Z,13Z,16Z)) | 2.28 | 2.27E-02 | 2.31 | Up | 1.000 |
|  | PS(20:5(5Z,8Z,11Z,14Z,17Z)/18:3(6Z,9Z,12Z)) | 2.49 | 2.02E-02 | 0.37 | Down | 1.000 |
|  | Sambutoxin | 3.33 | 3.41E-02 | 0.19 | Down | 0.938 |
|  | Linoleamide | 2.45 | 8.23E-03 | 0.33 | Down | 1.000 |
|  | Ginsenoside Rg3 | 4.96 | 2.79E-02 | 0.04 | Down | 1.000 |
| Organic acids and derivatives | Gluten exorphin C | 2.63 | 9.86E-03 | 2.74 | Up | 1.000 |
|  | Dibutyl malate | 1.91 | 8.42E-04 | 0.44 | Down | 1.000 |
| Organic nitrogen compounds | Dehydrophytosphingosine | 2.44 | 1.08E-03 | 0.33 | Down | 1.000 |
| Organic oxygen compounds | Nervonoylacetone | 2.87 | 1.26E-04 | 3.77 | Up | 1.000 |
|  | 4,6-Pentacosanedione | 1.89 | 2.13E-02 | 2.33 | Up | 0.938 |
| Organoheterocyclic compounds | Pipotiazine | 2.41 | 1.21E-02 | 2.51 | Up | 0.938 |
|  | 2-(4-Methyl-5-thiazolyl)ethyl octanoate | 2.51 | 3.45E-02 | 2.39 | Up | 0.938 |
|  | 14alpha-Hydroxypaxilline | 2.93 | 5.35E-03 | 3.28 | Up | 1.000 |
|  | Diosbulbinoside D | 1.93 | 5.96E-03 | 0.47 | Down | 1.000 |

*Variable importance in the projection (VIP) was acquired from OPLS-DA with a threshold of 1.0.

†P-values were corrected by Benjamini-Hochberg method with P < 0.05.

‡FC (Fold change) was obtained from the arithmetic mean values of each group. FC > 2 suggests a relatively higher concentration present in model cell while FC < 0.5 represents a lower concentration compared with the controls.
